# Barriers to implementation of nursing process in South Gondar Zone Governmental hospitals, Ethiopia

**DOI:** 10.1016/j.heliyon.2021.e06341

**Published:** 2021-03-01

**Authors:** Shegaw Zeleke, Demewoz Kefale, Worku Necho

**Affiliations:** a Department of Adult Health Nursing, College of Health Sciences, Debre Tabor University, Debre Tabor, Ethiopia; b Department of Pediatric and Child Health Nursing, College of Health Sciences, Debre Tabor University, Debre Tabor, Ethiopia; c Department of Maternity Nursing, College of Health Sciences, Debre Tabor University, Debre Tabor, Ethiopia

**Keywords:** Nurse, Nursing process, Implementation, Barriers

## Abstract

**Background:**

The nursing process is a global concept, which forms the foundation of nursing as a profession. The use of the nursing process in most hospitals is lagging despite all the efforts of nursing professionals to implement it. The nursing process is dynamic and it is used in clinical practice worldwide to deliver quality-individualized care to patients.

**Objective:**

This study assessed barriers to the implementation of the nursing process among nurses working at South Gondar Zone Governmental Hospitals, North Central Ethiopia, 2019.

**Methods:**

Institutional based cross-sectional study was carried out. By using Census about N = 249 nurses were recruited. Data was collected through self-administered questionnaires. The data were entered into Epi data version 3.1 and analyzed using statistical package for social sciences (SPSS) version 23.0. Descriptive statistics such as measurements of central tendency and inferential statistics multiple logistic regression, 95% CI, and p-value ≤ 0.05 was used. The study was approved by Debre Tabor University college of health sciences ethics and research committee.

**Results:**

A total of N = 249 study subjects participated with the response rate of 241 (96.4%). The mean age (SD±) of respondents' was 29.9 ± 7.2. About two-third of 146 (60%) nurses had poor knowledge of nursing process implementation. Three fourth 180 (74.7%) of nurses were implementing the nursing process. Nurses with sufficient information to NP were 2.45, nurses who have adequate skills to NP were 2.43, and nurses who have good knowledge were 2.24 times more likely to implement the nursing process than the opposite. No enough motivation to use NP 137 (56.8%), no follow-up by authority 141 (58.5%), no enough time for applying NP 145 (60.2%), no specific training for applying NP 173 (71.8%) and shortage of nurse staffs for nursing NP implementation 187 (77.6%) are also factors which affects nursing process implementation.

**Conclusion:**

Based on this study only three fourth of the nurses were implementing the nursing process. For poor and non-implementation of nursing process different hindering factors were identified. Such as; shortage of time, lack of training, lack of knowledge, unrecognized by authority, no enough motivation, lack of cooperation b/n professionals, engaging in other manual tasks and unrelated tasks, unclear and poor job descriptions, work overload and poor payment for the profession were the major barriers for NP implementation**.** Therefore, there need to be strengthen national policy frameworks and interventions aimed at improving nursing process training and implementationin in the clinical settings.

## 1 Introduction

The nursing process (NP) is a systematic method that utilizes scientific reasoning, problem-solving, and critical thinking to direct nurses in caring for patients effectively [1]. The use of the nursing process in most hospitals is lagging despite all the efforts of nursing professionals to implement it [2]. NP is a widely accepted scientific method to guide procedures and quality nursing care [3].

Nursing process implementation in most hospitals especially in low and middle-income countries reportedly remains a challenge despite efforts being made [4]. Ethiopia being in this category of low-income countries is not free from these challenges. There is a need to expand nursing process implementation in the clinical settings, which translates to improved quality of patient care. Ethiopia has been achieved the minimum WHO recommendation of 1 nurse per 5000 population [5, 6].

Despite nursing process adoption as a framework of delivering quality nursing care practically, nurses find some barriers in their daily implementation of NP in health institutions contributing to poor quality health care (QHC) [7, 8, 9, 10, 11]. Applying the nursing process across the board requires an understanding of barriers affecting its utilization [12]. However, despite its benefits, 65% of nurses working at Central and Northwest Zone Tigray Region hospitals are not implementing nursing process [13] and also the overall implementation of nursing process at Addis Ababa hospitals, Felege Hiwot referral hospital, Debretabor general hospital and Finoteselam general hospitals is 52.1%, 49%, 68%, and 69% respectively [9, 11]. This led to poor patient care and outcome. Poor quality health care in turn leads to increased morbidity and mortality rates in our health care institutions. This prompted the researchers to study barriers that hinder nurses from implementing NP at South Gondar Zone hospitals, Ethiopia.

## 2 Methods

### 2.1 Study area and period

The study was carried out in South Gondar Zone Governmental Hospitals which is found in South West Gondar, Amhara region, North Central Ethiopia. The capital city of South Gondar Zone is Debre Tabor which is located 50 km east of Lake Tana, 100 km from the capital city of the Amhara region, Bahirdar, and 666 km from the capital city of Ethiopia, Addis Ababa. The study was conducted from February 1^st^ –30/2019.

### 2.2 Study design

Institutional based cross-sectional study design was used.

### 2.3 Study population

All nurses (N = 249) working in South Gondar Zone Governmental hospitals were the total population for the study.

### 2.4 Inclusion criteria

○ Nurses who were working in South Gondar Zone Governmental Hospitals and enrolled during data collection time
○ Nurses who had at least greater than 6 months working experiences

### 2.5 Exclusion criteria

○ Nurses who were given free service
○ Nurses who were absent during data collection time

### 2.6 Sample size determination

Census was used because the study population was small N = 249.

### 2.7 Data quality assurance

The questionnaire was checked thoroughly for its objective and variable based before it was distributed to the respondents. Prior to the actual study period pretest was done on 12 (5%) nurses who were working in Addis Alem hospital, Bahirdar. One day training was given to the data collectors and the supervisors on the objective, the relevance of the study, confidentiality of information, respondent's right, and informed consent.

### 2.8 Study instruments

Structured self-administered questionnaires adapted from previously published literatures [7, 14] were used.

## 3 Operational definition

• **Nurse**: any person employed in the hospital to provide nursing services and is directly under the director of nursing services irrespective of his or her level of training or specialization.
• **Good knowledge of the nursing process**: Those respondents who were scored above and equal mean score of the knowledge questions.
• **Poor knowledge of the nursing process**: respondents who were scored below the mean score of the knowledge questions.

### 3.1 Ethics approval and consent to participate

The study was approved by Debre Tabor University college of health sciences ethics and research committee. This study was conducted in accordance with the Declaration of Helsinki: each study participant was well informed about the aim of the study, benefits and risks; informed written consent was secured from study participants; study participants confidentiality was maintained; no personal identifiers were used in the data collection questionnaire, and codes were used in place of them; the recorded data were not accessed by a third person, except the researcher.

### 3.2 Data collection, cleaning, and entry

Questionnaires were administered by four lecturers and two supervisors. Upon nurses filling the questionnaires, the data collector and supervisors were checked for completeness. Data was entered into the computer in preparation for data analysis using Epi Data version 3.1.

## 4 Data analysis and presentation

Analysis of data was done using SPSS version 23.0. Descriptive statistics such as measurements of central tendency (mean, mode, median) were used. Inferential statistics, such as the test of significance and coefficient correlations were used to compare variables. Files were presented with text and frequency tables.

## 5 Results

### 5.1 Socio-demographic characteristics of the study participants

In this study, a total of N = 249 study participants participated with the response rate of 241 (96.4%). The mean age (SD±) of respondents' was 29.9 ± 7.2. Almost two-third of 162 (67.2%) the respondents were in the age group of 20–29 years and half 120 (49.8%) of them were single in their marital status. About three fourth 181 (75.1%) of the respondents were BSc nurses in their academic qualification (Table 1).

**Table 1:** Distribution of socio-demographic characteristics of respondents among nurses working at South Gondar zone Governmental hospitals, Ethiopia, 2019.

Variables	Category	Frequency	Percent (%)
Age of respondents in year	20–29	162	67.2
	30–39	53	22.0
	40–49	17	7.1
	50–59	9	3.7
Sex of respondents	Male	148	61.4
	Female	93	38.6
Marital status	Married	111	46.1
	Single	120	49.8
	Divorced	8	3.3
	Widowed	2	.8
Academic qualification	Diploma	60	24.9
	BSc	181	75.1
Work experiences in year	below 5	114	47.3
	5–10	86	35.7
	11–15	13	5.4
	16–20	6	2.5
	21–25	13	5.4
	26–30	5	2.1
	above 30	4	1.7
Working unit	Medical ward	35	14.5
	Surgical ward	49	20.3
	Pediatric ward	45	18.7
	Obstetrics and Gyne ward	11	4.6
	Accident and emergency	29	12.0
	Psychiatry	4	1.7
	OPD	45	18.7
	Intensive care unit	23	9.5

### 5.2 Knowledge of nurses about nursing process

Among nurses who were participated in study 202 (83.8%) of nurses said patient priority identification is easy with NP, 146 (60.6%) of nurses correctly sequences NP steps but only 90 (37.3%) of nurses correctly write at least one nursing diagnosis (Table 2).

**Table 2:** Knowledge of nurses about nursing process implementation among nurses working at South Gondar zone Governmental hospitals, Ethiopia, 2019.

Variables	Category	Frequency	Percent (%)
Ever trained nursing process	Yes	126	52.3
	No	115	47.7
NP training makes competent for nursing practice	Yes	96	74.4
	No	33	25.6
Patient priority identification is easy with NP	Yes	205	85.1
	No	36	14.9
NP provides quality patient-centered care	Yes	202	83.8
	No	39	16.2
NP should be implemented for every patient	Yes	180	74.7
	No	61	25.3
NP is tedious	Yes	172	71.4
	No	69	28.6
Correctly sequences NP	Yes	146	60.6
	No	95	39.4
Correctly write at least one nursing diagnosis	Yes	90	37.3
	No	151	62.7

About two-third 146 (60%) of nurses had poor knowledge on nursing process implementation and three fourth 180 (74.7%) of nurses were implementing nursing process in south Gondar Zone governmental hospitals, Ethiopia.

### 5.3 Barriers for nursing process implementation

This study identifies different barriers which affect nursing process implementation. Based on the identified barriers; no enough motivation to use NP 137 (56.8%), no follow-up by authority 141 (58.5%), no enough time for applying NP 145 (60.2%), no specific training for applying NP 173 (71.8%) and shortage of nurse staffs for nursing NP implementation 187 (77.6%) were some of the listed barriers for nursing process implementation (Table 3).

**Table 3:** Barriers which hinders nursing process implementation among nurses working at South Gondar zone Governmental hospitals, Ethiopia, 2019.

Variables	Responses	
	Yes	No
	N (%)	N (%)
Lack of sufficient information to NP	136 (56.4)	105 (43.6)
No belief for patient care using NP	109 (45.2)	132 (54.8)
No enough motivation to use NP	137 (56.8)	104 (43.2)
Inadequate skill for NP application	115 (47.7)	126 (52.3)
No interest in using NP	111 (46.1)	130 (53.9)
Lack of cooperation between nurses	122 (50.6)	119 (49.4)
Repetitious replacement of nurses	136 (56.4)	105 (43.6)
No format to write NP	81 (33.6)	160 (66.4)
No follow-up by authority	141 (58.5)	100 (41.5)
No enough time for applying NP	145 (60.2)	96 (39.8)
No attention to NP importance	148 (61.4)	93 (38.6)
No specific training for applying NP	173 (71.8)	68 (28.2)
Lack of education about NP principles by authority	150 (62.2)	91 (37.8)
Peers influence NP implementation	131 (54.4)	110 (45.6)
Family influence NP implementation	97 (40.2)	144 (59.8)
Cultural belief influence NP implementation	91 (37.8)	150 (62.2)
Hospital administration recognize NP as a framework of Nursing care	146 (60.6)	95 (39.4)
Hospital administration support NP implementation	130 (53.9)	111 (46.1)
Hospital management monitor NP implementation	137 (56.8)	104 (43.2)
Hospital management recognizes staff for applying NP for pt. care	152 (63.1)	89 (36.9)
NP is part of performance appraisal	140 (58.1)	101 (41.9)
Hospital rules guides NP implementation	158 (65.6)	83 (34.4)
There is enough qualified nurse to apply NP	139 (57.7)	102 (42.3)
Shortage of nurse staffs affects NP implementation	187 (77.6)	54 (22.4)

### 5.4 Multivariate analysis result of factors affecting NP implementation

In the bivariate analysis, 10 variables have met the criteria p-value ≤0.2 to use multivariate analysis. Four variables were significantly associated with a P-value of ≤0.05 at a 95% confidence interval. When adjusting all other factors; nurses who have sufficient information to NP were 2.45 times more likely to implement the nursing process than nurses who lack sufficient information to NP (AOR = 2.45, 95% CI = 1.16, 5.12. Nurses who have adequate skill to NP were 2.43 times more likely to apply the nursing process than nurses with the inadequate skill (AOR = 2.43, 95% CI = 2.53, 4.42). Nurses who have attention to NP importance were 1.75 times more reluctant to implement the nursing process than nurse's lacks attention to NP importance (AOR = 1.75, 95% CI = 1.79, 3.86). Knowledge of nurses was the most predictor to perform nursing process; nurses who have good knowledge were 2.24 times more likely to implement the nursing process than nurses with poor knowledge of the nursing process (AOR = 2.24, 95% CI = 1.14, 4.39) (Table 4).

**Table 4:** Logistic regression analysis for nursing process implementation among nurses working at South Gondar zone Governmental hospitals, Ethiopia, 2019.

		Nursing process implementation		OR, 95% CI	
		No	Yes	COR	AOR
Sex of respondents	Male	34	114	1	1
	Female	27	66	0.729 (0.04,1.31)	0.78 (0.43,1.43)
Lack of sufficient information to NP	Yes	46	90	1	1
	No	15	90	3.07 (1.59,5.89)∗	2.45 (1.16,5.12)∗
Not enough motivation to use NP	Yes	44	93	1	1
	No	17	87	2.42 (1.28,4.56)∗	1.12 (0.48,2.70)
Inadequate skillfulness for NP	Yes	38	77	1	1
	No	23	103	2.21 (1.22,4.01)∗	2.43 (2.53,4.42)∗
No interesting in using NP	Yes	34	77	1	1
	No	27	103	1.68 (0.94,3.02)	1.00 (0.49,2.03)
Repetitious replacement of nurses	Yes	41	95	1	1
	No	20	85	1.83 (1.01,3.37)∗	1.29 (0.63,2.67)
No attention to NP importance	Yes	47	101	1	1
	No	14	79	2.63 (1.35,5.11)∗	1.75 (1.79,3.86)∗
No specific training for applying NP	Yes	49	124	1	1
	No	12	56	1.84 (0.91,3.74)	1.29 (0.58,2.58)
Not enough time for applying NP	Yes	41	104	1	1
	No	20	76	1.49 (0.81,2.76)	0.92 (0.45,1.86)
knowledge level	Poor knowledge	45	101	1	1
	Good knowledge	16	79	2.20 (1.16,4.18)∗	2.24 (1.14,4.39)∗

Note: ∗p value ≤0.05, COR = Crude odds ratio, AOR = Adjusted odds ratio, OR = odds ratio, CI = Confidence interval.

## 6 Discussion

According to this research result, the prevalence of nursing process implementation was 74.7%. This result is much more than nursing process implementation prevalence in Kenya 33.1% [20], Central and Northwest zones Tigray Region hospitals 35% [13], Addis Ababa hospitals 52.1% [16], Felege Hiwot Referral hospital 68%, Finoteselam general hospitals 69% and Debretabor general hospital 49% [16]. This discrepancy might be due to this research finding is not an observational study. The other reason may be time of the study; now a day nursing process implementation is one of the standards for the evaluation of hospitals. From barriers which hinder nursing process implementation 62.2% of nurses were agreed with no enough time for application of NP. This finding is similar to research done at Nairobi hospital 68.2% of nurses agreed that barriers related to nursing process implementation are related to lack of time to implement and NP is being time-consuming [17]. This similarity may be due to developing countries especially African's share similar workload to the nurses which they can't fulfill WHO nurse to patient ratio standards. The above research finding is also supported by findings in Vienna and South Africa. [14, 18] lack of adequate time, poor nurse-patient ratio, and high patient turn over hinders nursing process implementation. About two third of (61%) nurses have poor knowledge towards nursing process implementation. This result is congruent with research done at Tigray region about three fourth (77%) of nurses had moderate and poor knowledge. And knowledge is the major predictor for nursing process implementation which is supported by Yildirim B. and Queiroz A. research findings [19, 20]. Our research result showed that nurses who have good knowledge were 2.2 time s more likely to implement the nursing process than nurses who have poor knowledge. This research finding is supported by other research findings done at Addis Ababa, Ethiopia nurses who have good knowledge 38.9 times more likely to implement the nursing process than nurses who have poor knowledge [15]. The above finding is also comparable and supported by research done in Tehran; poor equipment, staff shortage, non -training of nursing staff, and unattractive service conditions can lead to non-implementation of the nursing process [21]. This indicates for the proper implementation of the nursing process trained and adequate nurse staff, clear job description, and recognition is not valid from the for mentioned institutions.

## 7 Conclusion

Based on this study only three fourth of the nurses were implementing the nursing process and about two-third of the nurses had good knowledge of the nursing process. Even if all nurses are expected to perform the nursing process some of the nurses were not practicing the nursing process. For poor and non-implementation of nursing process different hindering factors were identified. Such as shortage of time, lack of training, lack of knowledge, unrecognized by authority, no enough motivation, lack of cooperation b/n professionals, and being busy with manual activities. Even if nurses are practicing the nursing process, they are engaged in other manual tasks and unrelated tasks. And also the physicians consider the nurses as their servants. Especially, unclear and poor job descriptions, work overload, poor payment for the profession was the major hindrance for NP implementation. Nurses with sufficient information to NP, adequate skill to NP, attention to NP importance, and nurses who have good knowledge for NP were significantly associated with NP implementation. An appropriate and applicable nurse job description shall be applied in each health institution based on their competencies. And also the nursing practice act shall be applied accordingly to supervise and to manage nursing process implementation in the health institutions. There is a need to strengthen national policy frameworks and interventions aimed at improving nursing process training and implementationin in the clinical settings. Hospital management shall be recognized their activities and if necessary reward should be given. The hospitals shall be identified the need for nurses and better to fill their knowledge and skill gap with on-site or off-site training regularly. Nurses should be practicing their profession with the nursing process independently and collaboratively by considering patients as the central figure. Even if nurses have workload, there should do a nursing care plan for all patients and updated regularly using therapeutic nursing communication.

## Declarations

### Author contribution statement

Shegaw Zeleke: Conceived and designed the experiments; Analyzed and interpreted the data; Wrote the paper.

Demewoz Kefale and Worku Necho: Contributed reagents, materials, analysis tools or data; Wrote the paper.

### Funding statement

This research did not receive any specific grant from funding agencies in the public, commercial, or not-for-profit sectors.

### Data availability statement

Data included in article/supplementary material/referenced in article.

### Declaration of interests statement

The authors declare no conflict of interest.

### Additional information

No additional information is available for this paper.
